# Effects of Surface Passivation on Gliding Motility Assays

**DOI:** 10.1371/journal.pone.0019522

**Published:** 2011-06-03

**Authors:** Andy Maloney, Lawrence J. Herskowitz, Steven J. Koch

**Affiliations:** Department of Physics and Astronomy and Center for High Technology Materials, University of New Mexico, Albuquerque, New Mexico, United States of America; Dalhousie University, Canada

## Abstract

In this study, we report differences in the observed gliding speed of microtubules dependent on the choice of bovine casein used as a surface passivator. We observed differences in both speed and support of microtubules in each of the assays. Whole casein, comprised of α_s1_, α_s2_, β, and κ casein, supported motility and averaged speeds of 966±7 nm/s. Alpha casein can be purchased as a combination of α_s1_ and α_s2_ and supported gliding motility and average speeds of 949±4 nm/s. Beta casein did not support motility very well and averaged speeds of 870±30 nm/s. Kappa casein supported motility very poorly and we were unable to obtain an average speed. Finally, we observed that mixing alpha, beta, and kappa casein with the proportions found in bovine whole casein supported motility and averaged speeds of 966±6 nm/s.

## Introduction

Kinesin-1 (hereafter referred to as kinesin) is an ATPase that converts chemical energy to mechanical work. It travels along microtubules in one direction and can carry with it various cellular items [1]–[5]. In vitro motility studies use two different methods to investigate the kinesin and microtubule system. In one method, microtubules are fixed to a coverglass and individual kinesin motion are observed either by single-fluorophore tracking, or by attaching beads to kinesin [6]–[8]. The other method is a gliding motility assay where kinesins are fixed to a glass slide and microtubules flow on top of a layer of kinesin [9]–[11]. In the gliding motility assay, motility is sustained by first passivating the glass to prevent kinesin's motor domains from becoming inactive when interacting with untreated glass.

Passivation of glass can be done with bovine serum albumin (BSA) [9]–[11], bovine casein [12]–[16], a lot of kinesin [17], or other compositions [18]. Bovine casein is the typical surface blocker used, mainly because it works well at passivation and is inexpensive. Casein is a globular protein that does not have a known crystal structure [17]. Bovine casein is comprised of four major subgroups: α_s1_, α_s2_, β, and κ. Depending on the mammal the caseins come from, there exists different ratios of these globular constituents. For instance, bovine casein contains α_s1_+α_s2_>β>κ and human casein contains β>κ with only trace amounts of α_s1_ casein [19], [20].

How casein passivates a glass surface in order to support kinesin for the gliding motility assay is still not very well understood. However, some work has been done to try and elucidate how casein passivates glass surfaces. Ozeki et al. showed that two layers of casein form on the glass surface to help support kinesin for motility [12]. Verma et al. [17] also investigated how kinesin and casein interact depending on which casein constituent from bovine milk was used. In their study, they showed that the number of microtubules that landed on the kinesin surface was affected by the casein passivation. Hancock and Howard also showed that the number of microtubules that landed on the kinesin surface was dependent on the number of motor proteins adhered to the glass slide [21]. Building on these prior studies, we investigated whether the gliding speed of microtubules was affected by the type of casein used to passivate the glass slide.

## Materials and Methods

### Open data and open notebook science

Raw data and all open notebook entries regarding this experiment are publicly available [22].

### Microscopy and software

Experiments were conducted on an Olympus IX71 inverted microscope using an Olympus 60×1.42 NA PlanApo objective. Rhodamine fluorophores attached to tubulin were illuminated with a 100 W mercury lamp (attached to the microscope and attenuated by 94%) using a TRITC filter cube with Chroma's filter set 49005. The strong attenuation was to help reduce photobleaching and potential local heating of the sample. Image sequences were captured using custom LabVIEW software with an Andor Luca S camera. Data analysis was done with custom LabVIEW tracking software. A description of the tracking and camera software will follow in another paper, however, a link to the readme file for the software can be found here [22].

The microscope objective was held at a constant temperature using a polyimide film resistive heater, a temperature controller from TeTech, two 15 kΩ thermistors, and LabVIEW software. The design was very similar to the work done by Mahamdeh and Schäffer [23] and a complete description of our design can be found in the supporting information Text S1. Briefly, the objective was thermally isolated from the objective turret using a spacer. The control thermistor was attached to the objective near its base and the control circuit from TeTech monitored it and used the heater, placed directly above the thermistor, to heat the objective. The other thermistor was bonded to the top of the objective using thermal epoxy and was used as the temperature probe. This design can maintain a temperature to ±0.1°C. There are very few studies that indicate whether or not observation of the gliding motility assay was done with temperature stabilization or not. It has been shown that temperature does play a crucial role in obtaining stable data [10], [11] and we have also seen the effects of non temperature stabilization in our own data, see the supporting information Text S2. Due to the thermal connection with the mercury arc lamp, the entire microscope body heats up with a time constant of several hours. Without temperature stabilization, this caused a steady increase in gliding speed over the course of several hours [24].

### Flow cells

Gliding motility assays were performed using custom flow cells. A detailed description for the construction of the flow cells can be found in the supporting information Text S3. Briefly, a slide and cover slip were sandwiched together using double stick tape. The tape was positioned on the slide such that a channel of approximately 10 µL volume was formed. After trimming excess tape, a cover slip was placed over the channel and pressed to ensure proper adhesion to the tape. Once the flow cell had been prepared for observation, nail polish was used to seal the open ends. We have not observed any adverse affects to the assay by using nail polish as the sealant nor have we tried other brands than the one we use regularly, which is made by NYC.

### Buffers and solutions

The preferred solution to conduct gliding motility assays in is usually called BRB80 [25], and is also known as PEM. We prepare a 10x PEM solution containing: 800 mM PIPES (Sigma 80635), 10 mM EGTA (Sigma 80635), 10 mM MgCl_2_ (Sigma M1028) and pH-ed to 6.89 using approximately 1.25 M NaOH (Fisher S318) in 18.2 MΩ-cm water. The amount of NaOH was approximate since each solution of PEM was pH-ed to 6.89. Böhm [11] showed that gliding speed was affected by both the pH and the ionic strength of the solution the motors were in. In an effort to reduce as many variables as possible for speed measurements, we chose to maintain the pH of our PEM buffers at exactly 6.89 and vary the amount of NaOH necessary to achieve this pH. All buffers and solutions were prepared in 18.2 MΩ-cm water produced with a Barnstead EasyPure RoDI system. The 10x PEM stock solution was diluted by a factor of 10 at the time of experiments. The PIPES and EGTA we used were acid forms of the chemicals to allow us the ability to choose different cations to add to the solution in the form of NaOH or KOH. Once pH-ed, the PEM solution was passed through a 0.2 µm syringe filter and aliquoted in 1 mL screw top vials and stored at 4°C.

A mixture of the α_s1_- and α_s2_-caseins purified to 70%, β-casein purified to 98%, and κ-casein purified to 70% were purchased from Sigma (C6780, C6905, and C0406 respectively). Each casein component was dissolved in PEM under constant stirring. α-casein took approximately 60–80 minutes of constant stirring before no more precipitate was visible in solution, β-casein took approximately 30–40 minutes, and κ-casein required 15–20 minutes, all at room temperature. Visible whole casein (Sigma C7078) precipitate remained in solution if no heat was applied. Whole casein is not susceptible to thermal denaturation and was not affected by moderate heating [26]. Heating PEM to 60°–80°C while stirring in whole casein, caused the visible precipitate to be dissolved in PEM. We used a condenser to prevent evaporation of the buffer while heating. See the supporting information Text S4 for a detailed description for adding whole casein to PEM. All casein solutions were reconstituted to 1.0 mg/mL in PEM. After all the casein was dissolved in solution, it was stored at 4°C in convenient aliquots with no additional filtering. Casein solubility is a complicated function of other casein constituents in solution [27], [28], temperature [29]–[31], genetic variants [32], ionic strength and types of salts in solution [32], calcium ion concentration [27], [28], [32], and pH [33]. Casein solubility measurements were not performed in this study.

Microtubules were polymerized from bovine tubulin purchased from Cytoskeleton. We used both unlabeled (TL238) and rhodamine-labeled bovine tubulin (TL331M), stored as lyophilized aliquots at −80°C. Tubulin was polymerized in PEM, with the addition of 1 mM GTP (Sigma G8877), an extra 1 mM MgCl_2_, and 6% (v/v) glycerol (EMD GX0185). The inclusion of an extra 1 mM MgCl_2_ was to ensure that the EGTA in PEM did not chelate all the magnesium ions from solution since tubulin polymerization requires magnesium ions [25]. Adding glycerol to the storage/polymerization solution speeds up microtubule polymerization [34]. Polymerization was carried out in a Thermo PCR Sprint thermocycler held constant at 37°C and incubated for 30 minutes using 29% rhodamine-labeled and 71% unlabeled tubulin. After 30 minutes, the microtubules were fixed and diluted by 200x with a solution of PEM with 10 µM Taxol (Cytoskeleton TXD01) and removed from the thermal cycler. Our typical polymerization volume was 1 µL. After the polymerization cycle was complete, we added 199 µL of our PEM plus Taxol solution to stabilize the microtubules. Taxol is not highly-soluble in water and must be suspended in DMSO (Sigma D2650). We reconstituted Taxol to a final concentration of 10 mM in DMSO and stored it in a Bionexus e•IceBucket at 3°C. Taxol can create crystals in aqueous solutions due to its low solubility. It also has a high affinity for free tubulin and or rhodamine dye molecules [35], [36]. This can cause Taxol crystals to appear to be fluorescent microtubules [37]. In order to reduce the prevalence of Taxol crystals, any solution containing Taxol was always prepared fresh and immediately before experiments. No stock solutions containing Taxol in an aqueous environment were prepared for future use. Polymerized microtubules were stored at room temperature and protected from ambient light until used in a motility assay. Storing polymerized microtubules at 4°C will cause rapid depolymerization [34].

Our kinesin was generously supplied by Dr. Haiqing Liu and was supplied to us in 20 µL aliquots at a concentration of 0.275 mg/mL kinesin. The kinesin is his-tagged, truncated kinesin-1 dmk401 [38], [39]; from drosophila and was expressed in E. coli. Kinesin was diluted to 27.5 µg/mL for each assay.

### Motility assays

Motility assays contained 10 µM Taxol, 1 mM Mg-ATP (Sigma A9187), 20 mM D-glucose (Sigma 49139), 2.5% (v/v) of an oxygen scavenging antifade system, and 5 µL of fixed polymerized microtubules for a total volume of 100 µL in PEM with no added casein. The kinesin home page [40], as well as Verma et. al. [17], suggest that the inclusion of casein to the motility solution will enhance the chances of a gliding motility assay to work properly. We have observed that adding casein to the motility solution caused microtubules to undergo non ideal motility, i.e. they moved in tight circles and ended up wrapping around themselves such that the microtubules looked like squiggles and were untrackable. In order to prevent such behavior, we do not include casein in our motility solution. Our antifade system was a dual enzymatic oxygen scavenging system that contained in the stock solution; 800 µg/mL glucose oxidase (Sigma G6641), 2000 µg/mL catalase (Sigma C9322) and 20% (v/v) of 2-mercaptoethanol, referred to as BME, (Sigma 63689). When diluted into the motility solution, there was 8 µg/mL glucose oxidase, 20 µg/mL catalyase and 0.5% (v/v) BME. Antifade cocktails were prepared in advance and stored in 5 µL aliquots at −20°C. We have noticed that the antifade system will remain viable at −20°C for only one week. Beyond one week of storage, the antifade solutions were disposed of and fresh aliquots were prepared when needed.

Flow cells were constructed and incubated at room temperature (usually between 24°C and 25°C) with the various caseins for 10 minutes. Kinesin was then diluted in PEM with 1 mM Mg-ATP and 0.5 mg/mL of the same casein used for the passivation in a ratio of 1∶10 kinesin:buffer for a final kinesin concentration of 27.5 µg/mL of kinesin in solution. The kinesin was introduced into the flow cell by fluid exchange and allowed to incubate for another 5 minutes. Finally, our motility solution was flowed into the cell that was then sealed with nail polish to prevent evaporation.

### Experiment and data collection

After sealing the flow cell, the slide was immediately placed on the microscope. Data was taken at 5 frames per second with each frame having an exposure of 100 ms and a EMCCD gain of 150. Multiple regions on the slide were exposed and images were collected for at least 20 regions of interest (ROIs) for each slide. Each ROI was exposed for approximately 2 minutes allowing the camera and computer to collect 600 total frames.

Data was then analyzed using customized LabVIEW (National Instruments, Austin, TX) automated microtubule tracking software. A detailed description of the tracking software will follow in another paper [41]. Briefly, microtubules were identified by NI Vision 7.1 image segmentation algorithms and the ends of microtubules were indentified via pattern matching algorithms. Tracking of a microtubule was stopped if it got too close to the edge of the field of view or, if it overlapped with another microtubule. Data were discarded for microtubules tracked for fewer than 100 consecutive image frames. Tracks were also discarded for microtubules with a segmented area less than 55 pixels. This filtering prevented tracking of microtubules that were either too small for the image recognition to precisely locate the ends of the microtubule or microtubule tracks that did not have enough data points for the subsequent speed analysis.

Automated tracking provided the x and y position with subpixel accuracy of the microtubule ends for each frame that was tracked. These time series data were smoothed with a sliding Gaussian window with a standard deviation of 2 seconds. Smoothed data points that were within 5 seconds of the beginning or end of the tracks were discarded in order to eliminate boundary effects on the smoothed data due to the window. Smoothing the position versus time data was necessary since the microtubule ends are never permanently attached to the kinesin surface and thus undergo transient Brownian motion. After smoothing and truncating, the instantaneous speed was calculated as the distance per time between consecutive frames.

Speed versus time data for all the microtubules in an individual ROI were then concatenated together and the most likely speed was extracted using a kernel density estimation (KDE) [42] with a Gaussian kernel of width 50 nm/s. The ROIs were 2 minutes long, had 600 images, and could have one to over 100 microtubules that needed to be tracked. We used a KDE method instead of a simple mean because we wanted to reduce our sensitivity to microtubule pausing or stalling, which was evident in many assays. We used a large kernel width to reduce sensitivity to possible speed changes due to number of kinesin motors or other causes [43]. The most likely speeds for individual regions were then plotted versus time to determine when the slide had reached thermal equilibrium with the objective. The initial 5 data points were removed for all data sets indicating that it took about 10 minutes for the slide to reach a stable temperature. Each assay; alpha, beta, kappa, whole, and mixed casein was repeated 3 separate times on different days and with different kinesin aliquots. When possible, the mean and standard error of the mean was computed for the three data points for a given assay time. Time differences of ±20 seconds were ignored for this calculation.

## Results and Discussion

### Results

Data was taken at 33.1±0.1°C as measured from the top thermistor on the objective. This temperature is well above the temperature the objective would reach due to long-term heating from the Hg lamp and was found to give consistent data. We observed (data not shown) that the closer the observation was to the boundaries of the flow cell, the slower the microtubule gliding speed was. We also observed that the propensity for depolymerization increased near the boundaries. In order to obtain consistent data and prevent depolymerization of the microtubules, gliding assays were observed in the center of the flow cell channel, except where otherwise noted. All images have had dead pixels removed by an interpolation function and have been false colored using ImageJ's green fire blue LUT using a custom LabVIEW 7.1 application.

**Figure 1:**
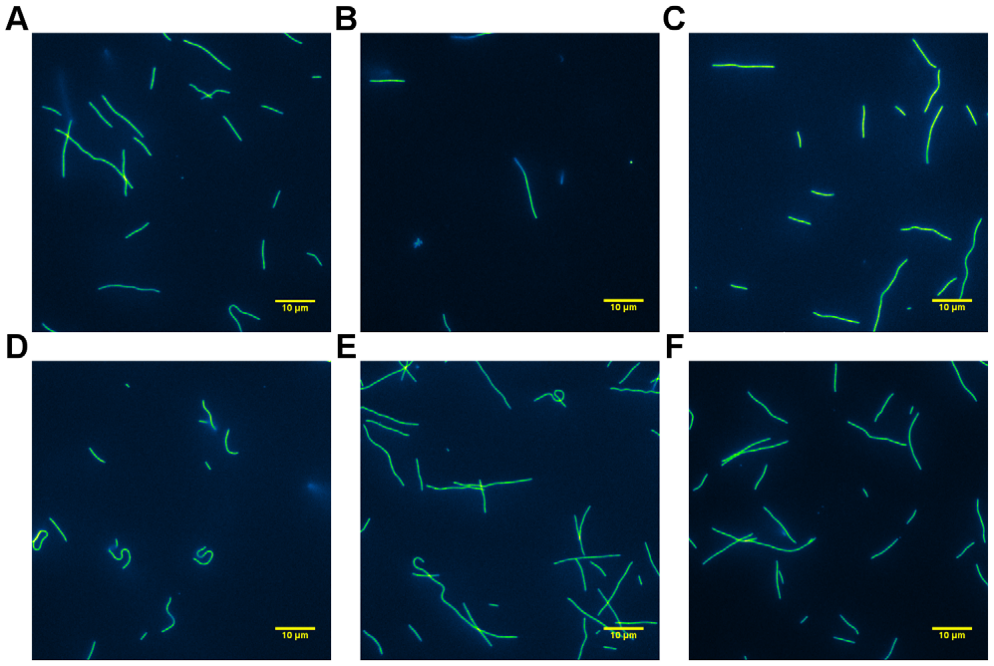
Observations of microtubules in flow cells passivated by different types of casein. Images have been false colored with ImageJ's Green Fire Blue LUT and have had dead pixels removed using an interpolation function. **A.** Alpha casein (Sigma C6780) passivation showed support for both long and short microtubules. **B.** Beta casein (Sigma C6095) passivation did not support motility very well and did not support shorter microtubules. **C.** Kappa casein (Sigma C0406) did support motility but only in limited regions of the flow cell. In other regions near the center of the channel **(D)** the microtubules were stuck to the slide and did not exhibit motility. **E.** Commercially available whole casein (Sigma C7078) is the standard for surface passivation for the gliding motility assay. It supported both long and very short microtubules. **F.** Mixed casein which was made from 49% α, 37% β, and 14% κ casein worked just as well as alpha casein and whole casein passivation in terms of number and sizes of microtubules exhibiting motility.

Bovine alpha casein (a mixture of α_s1_ 37% and α_s2_ 10%) constitutes approximately 47% of whole bovine casein [20]. This passivation was capable of supporting small microtubules and longer ones as can be seen in Figure 1A. When using alpha casein, the gliding motility assay worked every time except when we deemed the kinesin or antifade system to have lost its effectiveness for maintaining a gliding assay.

Bovine beta casein comprises approximately 35% of whole casein and visible precipitates took less time to dissolve in PEM as compared to alpha casein. Figure 1B shows that beta casein as a surface passivator was not ideal. It did not support smaller microtubules and did not in general have very many motile microtubules in any assay. The microtubules that were motile in this assay were typically quite long. Beta casein also caused the microtubule's minus and positive ends to detach from the kinesin surface while undergoing motility more often than the other passivation schemes. This caused errors in the tracking and thus did not give consistent data. However, it can be purified to better than 98% making this component of whole casein the purest commercially available. Another interesting phenomenon observed when using beta casein was that motile microtubules were not found in the center of the channel for the flow cell. Motile microtubules were observed, but they were always found off center to the flow cell channel. We do not know the reason for this, and it may depend on kinesin or casein concentration. We did not vary the input kinesin or casein concentration for these studies.

Kappa casein, compared to alpha and beta, is structurally very different. It is a glycoprotein and is thought to stabilize the casein micelle [20], [44], [45] by sterically hindering the aggregation of too many casein sub-micelles. It did not support motility in a very consistent manner as can be seen from Figure 1C&D. As was the case for beta casein, kappa casein did not support motility in the center of the channel of the flow cell. Stuck microtubules were found near the center of the flow cell and near the tape. However, between the boundaries of the flow cells and away from the center of it, there was motility. In the areas that motility existed, kappa casein was able to support motility of long and short microtubules with the exception of extremely short microtubules found to move only in the alpha or whole casein assays. The very short microtubules either remained stuck to the surface or exhibited motility for a very short period of time before going into solution. We also observed that kappa casein did a remarkable job of adhering microtubules to the slide much like how poly-L-lysine is used to fix microtubules to glass [46].

Whole bovine casein is the passivator of choice when doing gliding motility experiments. We found that whole bovine casein worked remarkably well for sustaining motility. Similar to the alpha casein passivation, whole casein worked every single time and gave consistent data. It was only when we deemed either the kinesin or the antifade system to have lost its effectiveness at maintaining microtubule gliding that the assay did not work. Of the 5 types of bovine casein solutions tested, whole casein required heat in order for visible precipitate to completely dissolve in PEM. See the supporting information Text S4 for a more thorough discussion of how we dissolve whole casein in PEM. We did not observe any adverse affects to motility by heating whole casein. Whole casein supported long and short microtubule motility as shown in Figure 1E.

Mixing whole casein from the individual constituents of alpha, beta, and kappa was also used as a surface passivator. The mixed whole casein consisted of 49% alpha casein, 37% beta casein and 14% kappa casein which was very similar to what Fox and McSweeney state as the casein components of bovine milk [20]. Mixing it was easy since each component was already in a PEM solution. The behavior of the mixed bovine whole casein was indistinguishable from the purchased whole casein or the alpha casein passivation. The number of microtubules and the varying lengths undergoing motility in the mixed casein passivation was very similar to those of the whole casein passivation as well.

**Figure 2:**
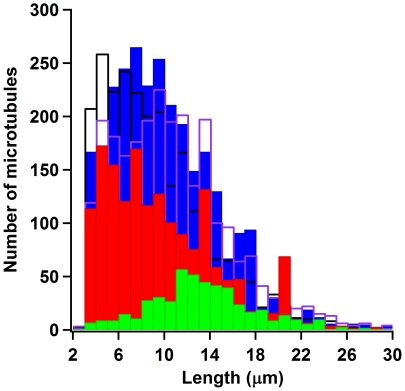
Relative microtubule length distributions. Filled blue bars are length calculations for the alpha casein passivation, filled green bars are for the beta casein passivation, filled red bars are for the kappa casein passivation, unfilled black bars are for the mixed casein passivation, and unfilled purple bars are for the whole casein passivation. Length measurements were performed only on tracked microtubules and are estimations from computing the Convex Hull perimeter on eroded images of microtubules.

Figure 2 shows a histogram of microtubule lengths. To obtain lengths, we used an erosion algorithm on binary images of only the microtubules that were tracked. We used a standard function in LabVIEW/Vision 7.1 called Skeleton L. After the erosion, we used a Convex Hull perimeter calculation also found in the LabVIEW/Vision 7.1 library of functions. We divided the Convex Hull perimeter by 2 to estimate the microtubule length. Two of the authors independently and manually estimated the length of several microtubules and found that usually the Convex Hull perimeter was in between both manually obtained values. We decided that this was sufficient for identifying large changes in MT length distributions, and so we did not further investigate the robustness or systematic errors in our length estimation. As can be seen in the figure, alpha casein (filled blue bars), whole casein (empty purple line bars) and mixed casein (empty black line bars) all were able to sustain motility with smaller microtubules 2–6 µm range. Beta casein (filled green bars) had significantly fewer microtubules that were tracked, however, those that were tended to be longer than the ones tracked in the alpha casein assay. Kappa casein (filled red bars) show that there were many more trackable microtubules than beta casein and significantly fewer than alpha casein. The smallest trackable microtubules using our algorithm are not smaller than 2 µm. Figure 2 shows that while in comparison to the other lengths, there are relatively few 2 µm microtubules in any of the assays, kappa casein had no microtubules that fell in the 2–3 µm range. There is, however, a larger number of kappa casein microtubules that were in the 20–21 µm range as compared to the other assays. Length measurements using this method are not optimized for precision, but this method does give a simple way to see the relative size distribution differences in the assays.

**Figure 3:**
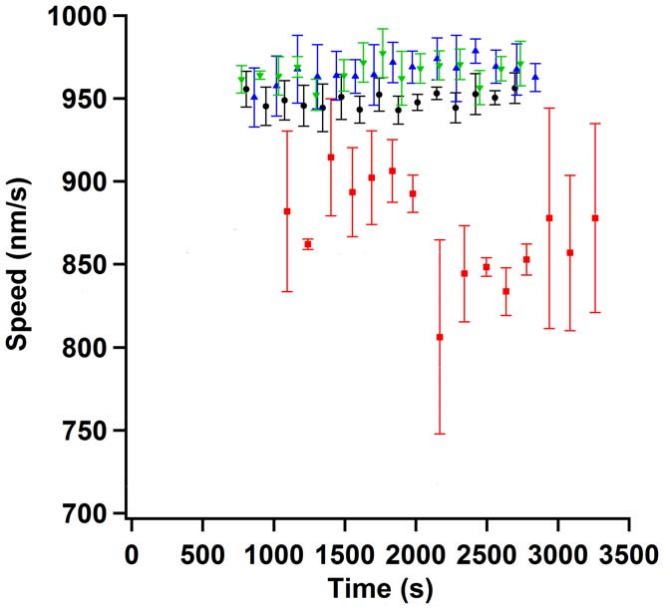
Speed versus assay time for four types of casein passivation. Black circles are alpha casein passivation, red squares are beta casein passivation, green up pointing triangles are whole casein passivation, and blue down pointing triangles are mixed casein passivation. Each data point is the mean from three different samples, taken at approximately the same assay time. Error bars represent the standard error of the mean. Alpha casein had the most consistent average speed measurements at 949±4 nm/s. Whole casein and mixed casein averaged to 966±7 nm/s and 966±6 nm/s respectively. Beta casein averaged to 870±30 nm/s.

**Figure 4:**
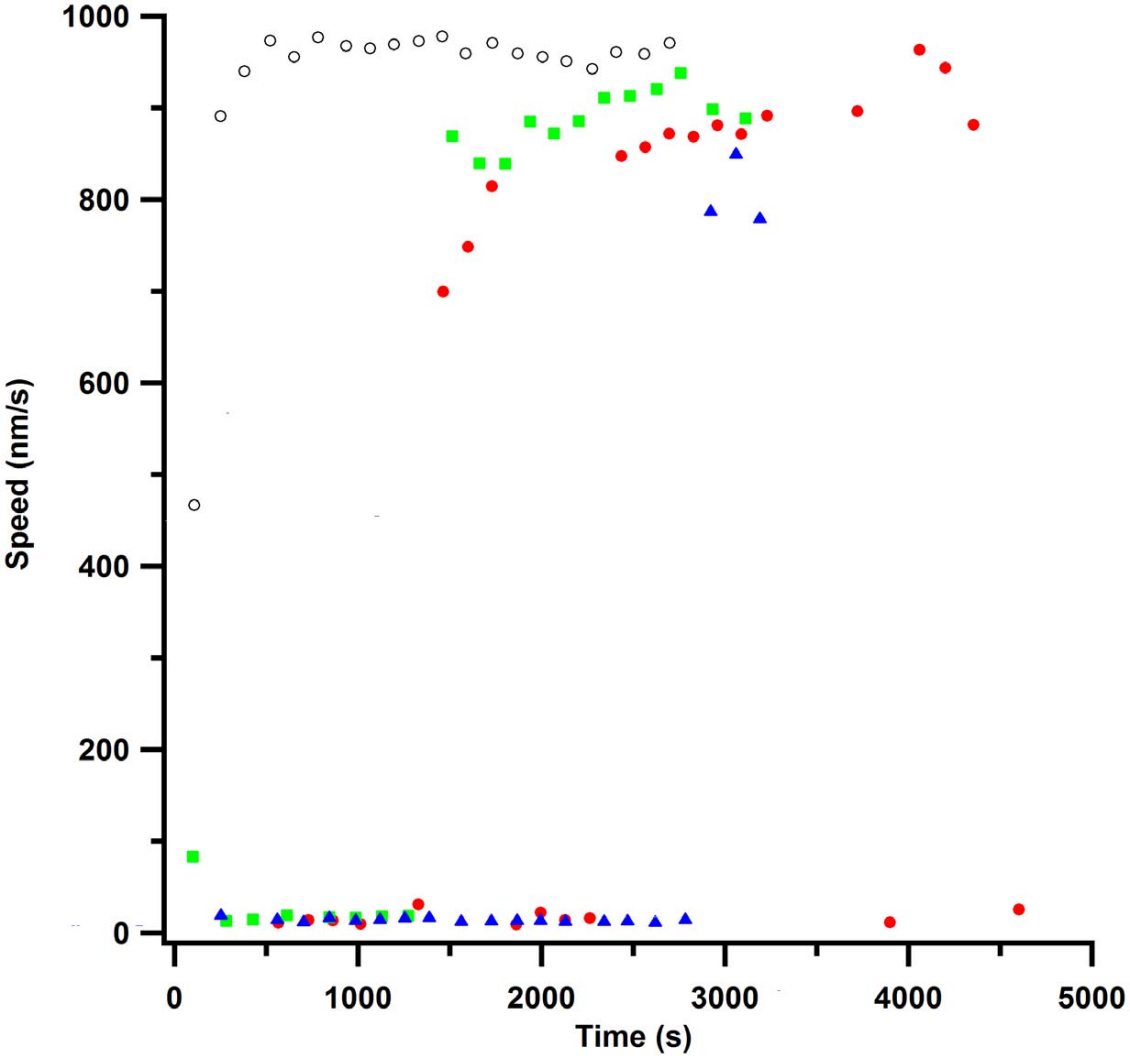
Gliding speed measurements with kappa casein passivation. Green squares, red circles, and blue triangles represent 3 different assays. As the figure shows, kappa casein was not the ideal surface passivator. There were many regions in the flow cell with no motility and other regions that showed inconsistent motility and at much slower speeds than was reported from alpha, whole or mixed casein passivations. The open black circles show a characteristic alpha casein assay for comparison. Note that the initial increase in speed (time less than 500 seconds) was due to the slide coming to thermal equilibrium with the objective. Because the three kappa casein samples appeared to have different speed distributions and because many regions of interest showed no motility, we did not attempt to compute a mean speed versus assay time, as was done in Figure 3 for the other caseins.

Figure 3 shows the mean speed measurements for 15 different regions of interest for the alpha, beta, whole and mixed casein assays. Each data point is the mean of a region of interest with SEM from three separate samples. The passivator that gave the most consistent speed was alpha casein. The mean speed and SEM from our alpha casein measurement was 949±4 nm/s. Purchased whole casein and mixed casein performed remarkably similarly and displayed average speed values of 966±7 nm/s and 966±7 nm/s respectively. Bovine beta casein performed poorly in comparison to alpha, whole, or mixed caseins and we measured the mean speed to be 870±30 nm/s. Figure 4 shows the observed speeds for kappa casein passivation. Since there were so many areas where no motility was observed in this assay, it was difficult to determine a mean speed measurement for each assay as was done in Figure 3. However, it does appear that when motile, the speeds were around 870–880 nm/s with kappa casein as the surface passivator. This was similar to how beta casein performed.

### Discussion

We observed that the more difficult it was to dissolve casein, the better it worked as a surface passivator. This may be a coincidence, or it may relate to the manner in which casein adsorbs to the glass. Whole casein was by far the most difficult to dissolve visible precipitate into PEM without heating. Alpha casein came in second, beta third and kappa fourth in terms of the time to dissolve completely in PEM at room temperature. Whole casein is approximately 50% alpha casein and seeing how well alpha casein performed as a surface passivator, we were not surprised that whole casein also performed well. The differences in measured speeds between alpha and whole casein could be a result of how kinesin was supported by the different caseins, or it could be due to differences in surface-coverage by the casein. It has been shown that using glass of differing hydrophilicity with whole casein passivation affects the activity of kinesin [47]. In this study, we observed a difference in speed due to the type of casein passivation.

Purchased whole and mixed whole casein had indistinguishable speeds, and no other observable differences. Mixed whole casein has an upper bound of 20% impurities in it. This amount was very similar to the amount of impurities alpha casein has in it yet, mixed whole casein performed exactly how purchased whole casein did. This similarity between mixed and purchased whole casein suggests that the speed difference between alpha and whole casein was not due to impurities but rather that it was due to how kinesin was supported by the casein micelles or, how casein interacts with the glass. To elucidate the effects, it would be prudent to measure the speeds for the various caseins as a function of casein and kinesin concentrations during incubation. We have not yet performed these experiments.

Beta casein would have been the most ideal protein to use as the surface passivator since one can purchase it to greater than 98% purity. Higher protein purity is advantageous for systematically producing devices that use kinesin and microtubules as sensors. However, beta casein did not perform very well. It had the least number of motile microtubules to track, it was not very reliable, and had a large distribution in speeds. It is possible, though, that varying other parameters such as kinesin concentration, or beta casein concentration could restore reliable motility. We have not yet attempted these experiments.

Kappa casein would be an attractive surface passivator just for its ease in dissolving in PEM. However, where motility exists in the flow cell was not consistent and it never occurs in the center of the channel where we have already observed the most consistent speed measurements from other assays. With kappa casein, we observed many long and stable microtubules permanently stuck to the surface. We are not sure if the sticking was caused from kinesin attaching to microtubules and then somehow being impeded from moving or, if there was actually no kinesin on the kappa casein surface and the microtubules are just attracted to the kappa casein or the glass.

Of the five types of bovine casein used to observe the gliding motility assay, alpha casein performed very well. It was the easiest of the three commercially available bovine casein constituents to dissolve in PEM and can be purchased at 70% purity. Most likely, the 30% contaminants are from other casein components. It performed well every time an assay was prepared and worked just as well as mixed and purchased whole casein.

### Conclusion

There are a wide variety of surface passivation strategies in the literature. Even for use of kinesin in gliding motility assays, where casein is the most commonly used passivator, there are many varieties to choose from. We found that alpha casein (Sigma C6780) was the most reliable passivator when following the most typical gliding assay protocol. We are currently using this as our passivator for studies on the effects of water isotope and osmotic stress on kinesin microtubule activity. We did not explore the parameter space of kinesin or passivator concentration or incubation temperature or time. Statistically designed experiments (DOE) may be an efficient method for optimizing the assay conditions for other casein varieties.

## Supporting Information

Text S1 **Objective heater.** Temperature stabilization of the objective was done with an objective heater. The following text describes the build to the objective heater.
(DOC)

Text S2 **Temperature data.** A description and graph showing the importance of temperature stabilization of the objective.
(DOC)

Text S3 **Flow cell construction.** Here we describe in detail how we made the flow cells used for this study.
(DOC)

Text S4 **Whole casein in PEM.** Here we describe in detail how we dissolved whole casein in PEM.
(DOC)
