# Supplementary material for: Effects of Surface Passivation on Gliding Motility Assays
Source: PLoS One. 2011 Jun 3;6(6):e19522. doi: 10.1371/journal.pone.0019522 (PMC3108588; doi:10.1371/journal.pone.0019522)

**Objective heater**

*Materials:*

Polyimide Film Heater Kit (Omega KH-KIT-EFH-15001)

Copper tape (McMaster-Carr 76555A712)

Power supply (TeTech PS-12-8.4)

Thermal spacer (Bioptechs RMS - 152019R)

Temperature controller (TeTech TC-48-20)

2x 15kΩ Thermistors (TeTech MP-2444 and MP-2996)

Bud Industries aluminum box (AC-404) and bottom plate (BPA-1591)

2 LEDs

2 switches

4 stainless steel standoffs (McMaster-Carr 92871A047)

Bumpers

*Step 1:*

Enclose the power supply in its own box. We added an *on* switch to turn on the power supply in the back and another *on* switch to turn on the power out to the banana connectors. The green LED indicates that the power supply is on and the yellow one indicates that power is being supplied out of the banana connectors. The bumpers are used to prevent the power supply from sliding around.


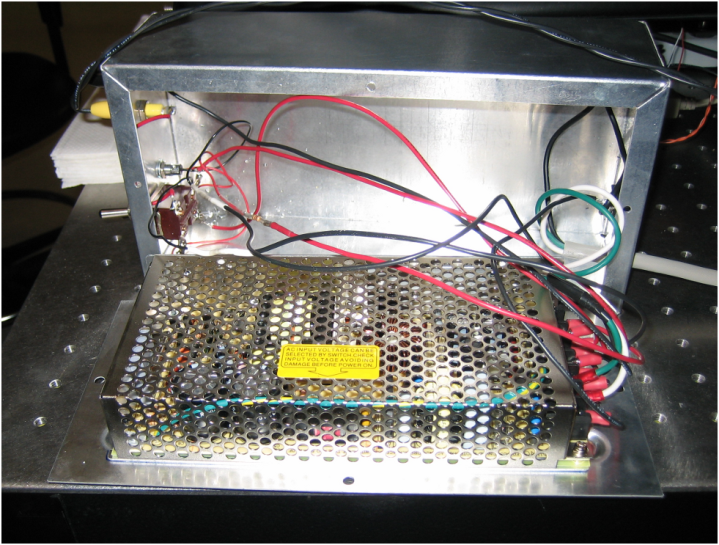


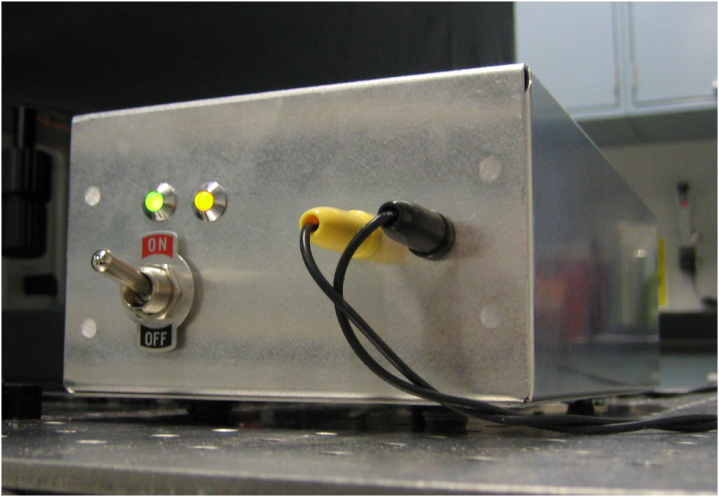


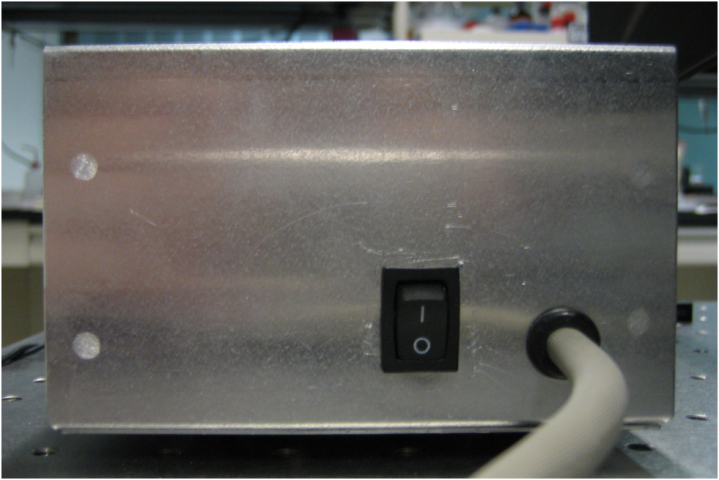


*Step 2:*

A break away box was added to the TeTech controller for easy connections for the thermistors and RS-232 cable. The blue+orange and white+orange banana connectors are for the thermistors. The red+green are for power to the flexible heater and the yellow+black are the power to the controller from the power supply. This is not necessary since the controller has screw terminals on it.


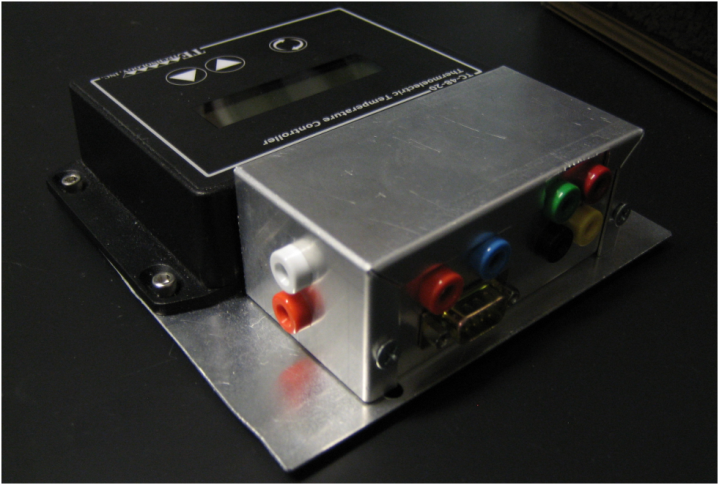


*Step 3:*

The Bioptech’s thermal spacer is between the objective and the turret. The control thermistor, MP-2996, is taped to the base of the objective with copper tape and wrapped around the objective completely one time. The flexible heater is then wrapped around the objective and alligator clips are used to supply power to it. The second thermistor, MP-2444, is epoxied to the top of the objective using thermal epoxy. The slide holder also has spacers on it to prevent direct contact of the slide to the aluminum holder.


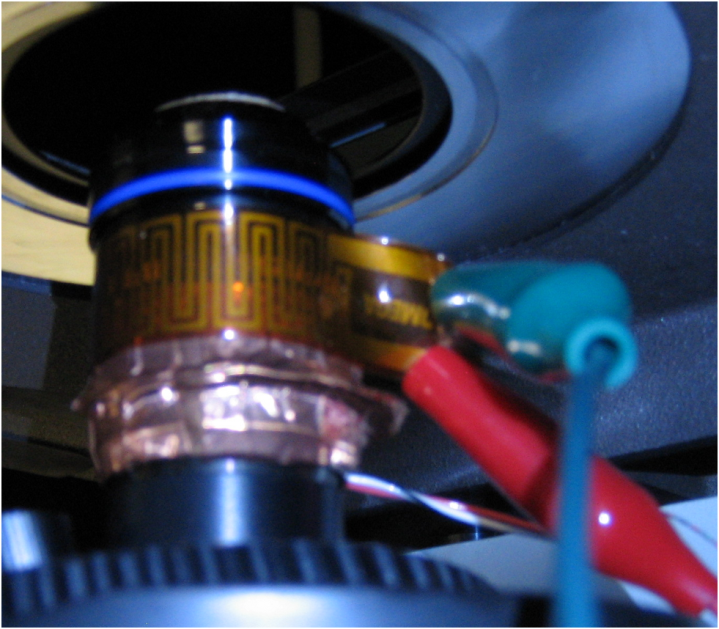


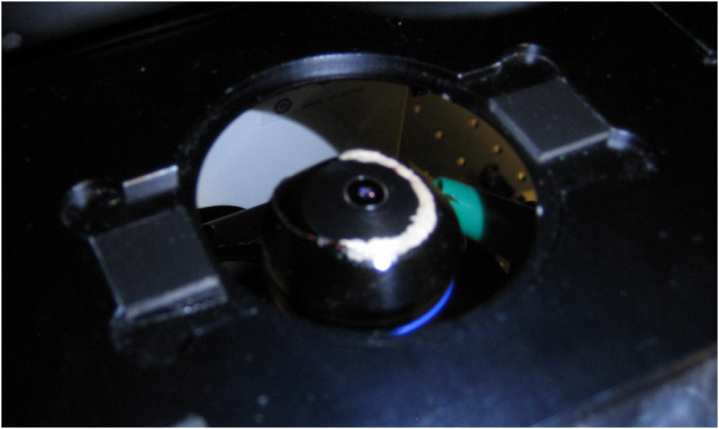


*Step 4:*

The supplied LabVIEW program from TeTech is used to monitor the power output of the controller as well as the temperature of the thermistors. It is capable of maintaining a constant temperature to ± 0.1°C.


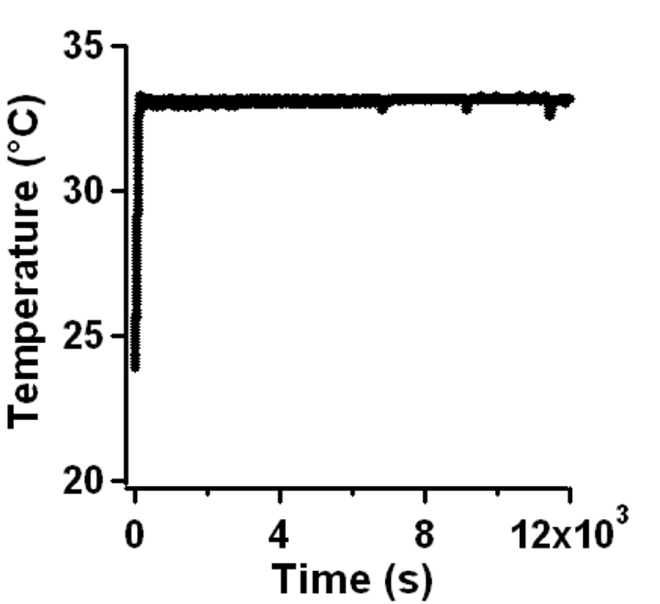

Supplement: Text S1 — Objective heater. Temperature stabilization of the objective was done with an objective heater. The following text describes the build to the objective heater. (DOC) [file pone.0019522.s001.doc]
