# Supplementary material for: Effects of Surface Passivation on Gliding Motility Assays
Source: PLoS One. 2011 Jun 3;6(6):e19522. doi: 10.1371/journal.pone.0019522 (PMC3108588; doi:10.1371/journal.pone.0019522)
Supplement: Text S2 — Temperature data. A description and graph showing the importance of temperature stabilization of the objective. (DOC) [file pone.0019522.s002.doc]

**Temperature data**

**
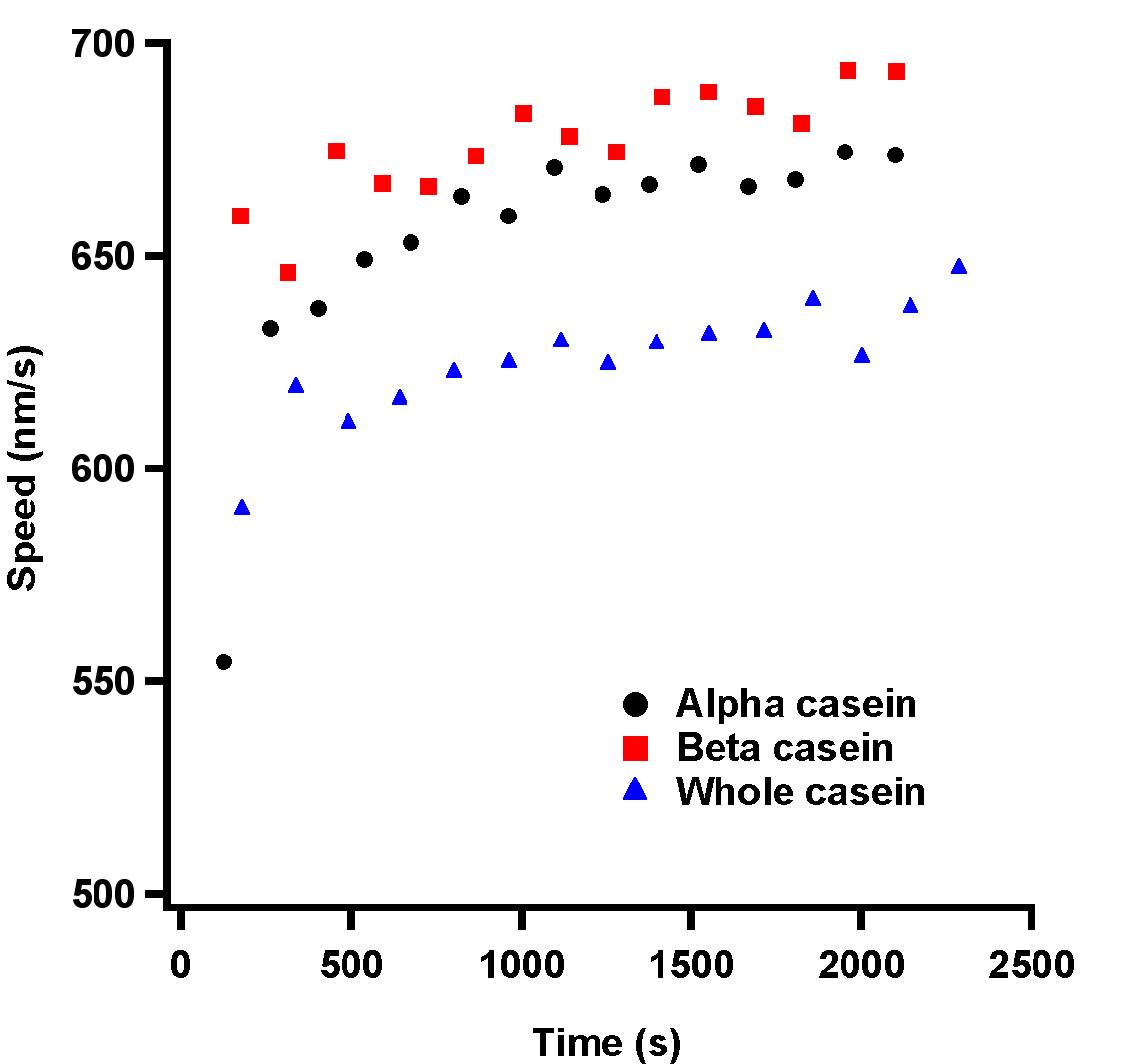

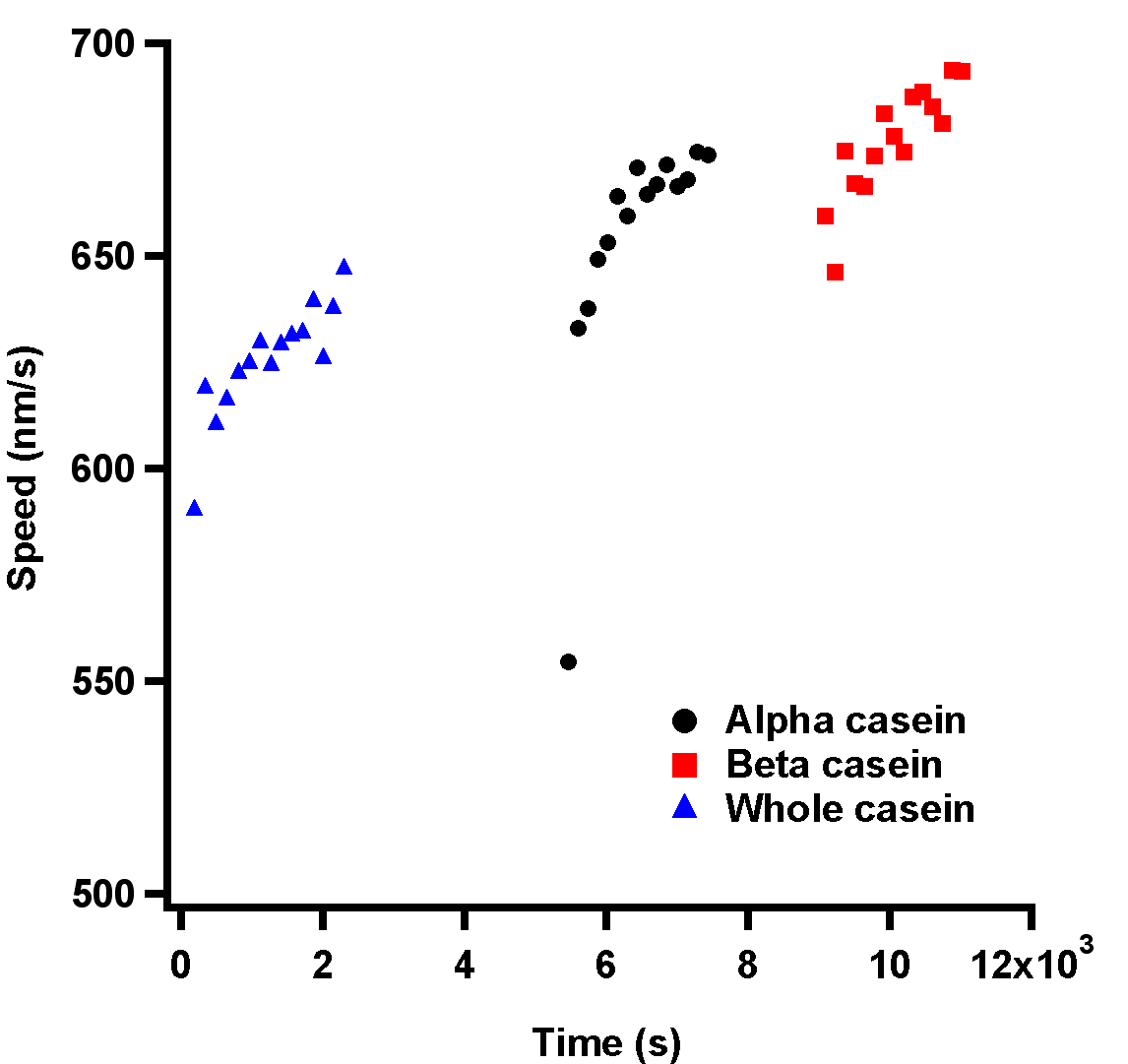
**

The graph to the left shows three assays with alpha, beta, and whole casein passivations. The data was taken before the objective was stabilized by the custom heater. All three assays were made in the same day and were done in the order: whole, alpha, and beta casein. The left graph shows each assay plotted with respect to the time each assay started. The graph to the right shows the same data, except each assay was graphed with respect to the time the first assay was conducted in the day. There are two important things to draw from this data. **1.** Each assay is prepared at room temperature and then placed on the microscope for observation. The increase in speed for each data set over time is due to the slide heating up. **2.** The overall increase in speed from assay to assay is due to the mercury lamp heating up the microscope over time. Another interesting point is that the measured speed at which the microtubules glide at is approximately 650 nm/s while the measured speed of the microtubules with temperature stabilization at 33.1˚C is 950 nm/s.
