# Supplementary material for: Effects of Surface Passivation on Gliding Motility Assays
Source: PLoS One. 2011 Jun 3;6(6):e19522. doi: 10.1371/journal.pone.0019522 (PMC3108588; doi:10.1371/journal.pone.0019522)
Supplement: Text S3 — Flow cell construction. Here we describe in detail how we made the flow cells used for this study. (DOC) [file pone.0019522.s003.doc]

**Whole Casein in PEM**

*Materials:*

Liebig condenser

50 mL Erlenmeyer flask

Stir bar

Stirrer/Hot plate

Fish tank pump

Tubing

Thermometer

Ice Bucket

Whole casein

*Step 1:*

Weigh out the appropriate amount of whole casein (Sigma C7078) in order to have 1.0 mg/mL whole casein in PEM. Add the casein to the PEM solution in the flask and add the stir bar.

*Step 2:*

Place the flask on the stirrer/hot plate and secure the condenser to the flask. We typically add support to the condenser by attaching it to chemistry clamps attached to a heavy base.


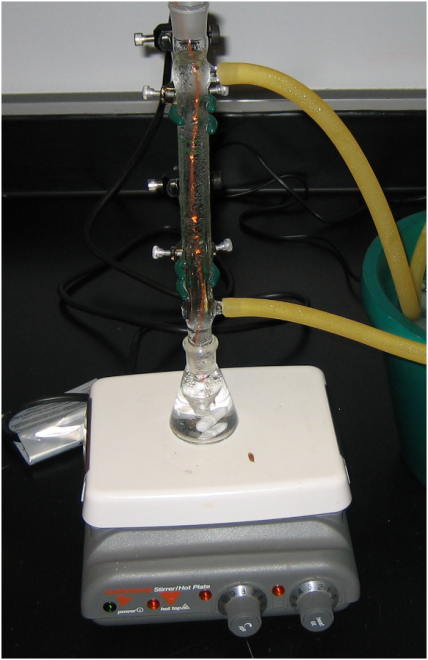


*Step 3:*

Attach the tubing to the condenser such that the pump is flowing ice water into the condenser from the bottom nipple and out of the condenser from the top nipple. The best way to prevent the pump from sucking air is to have water throughout the condenser/tube system before turning the pump on. To do this, use a syringe to suck out the air from the system.


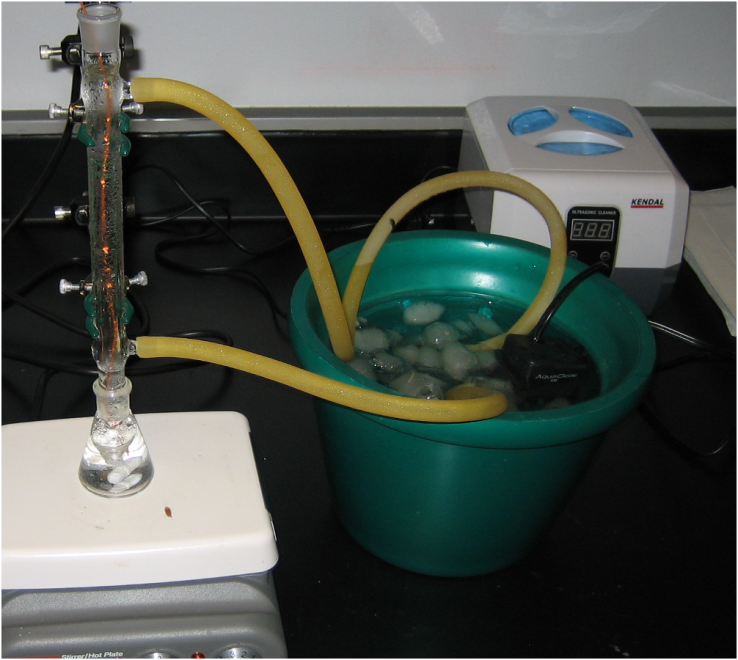


*Step 4:*

Since the condenser system is open to the atmosphere, we can put a thermocouple through it in order to test the temperature of the solution. Do so and turn on both the heater and the stirrer. Max out the speed of the stirrer and turn on the heater to a medium low setting. It should take approximately 15 minutes for the solution to reach a temperature of 60˚C. Once it has reached this temperature, turn off the hot plate and continue to stir the solution until all the casein has been dissolved.

*Step 5:*

The solution should be clear with foam on top of it. The foam is a good sign as it is an indicator that there is protein in the solution. To ensure that the protein stays in solution, seal the top of the flask with some Parafilm and let the solution sit overnight at 4˚C. Once the foam has settled, aliquot into 1 mL aliquots in screw top vials and continue to store at 4˚C.
