# Supplementary material for: Effects of Surface Passivation on Gliding Motility Assays
Source: PLoS One. 2011 Jun 3;6(6):e19522. doi: 10.1371/journal.pone.0019522 (PMC3108588; doi:10.1371/journal.pone.0019522)

**Flow cell construction**

*Materials:*

Slides VWR 48300-025

Cover slips VWR 48366-045

Box cutters

Double stick tape

Marker

Ruler

*Step 1:*

Marks are made on a table to indicate the spacing needed for the double stick tape. Typically, a 5-6 mm wide channel is large enough to hold approximately 10 μL volume.


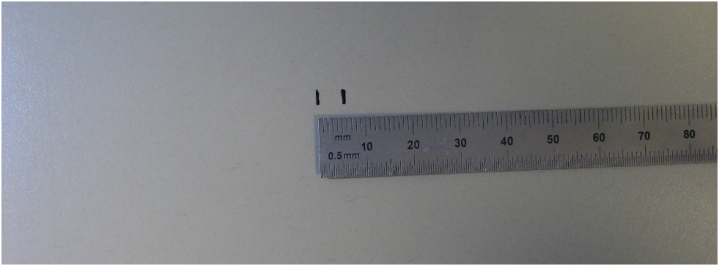


*Step 2:*

A slide is then centered over the marks and two pieces of double stick tape are placed parallel to them. To ensure that the channel formed between the pieces of tape stays clean, do not place the tape on the slide and lift it off. Once it touches the slide, that is where the tape will be.


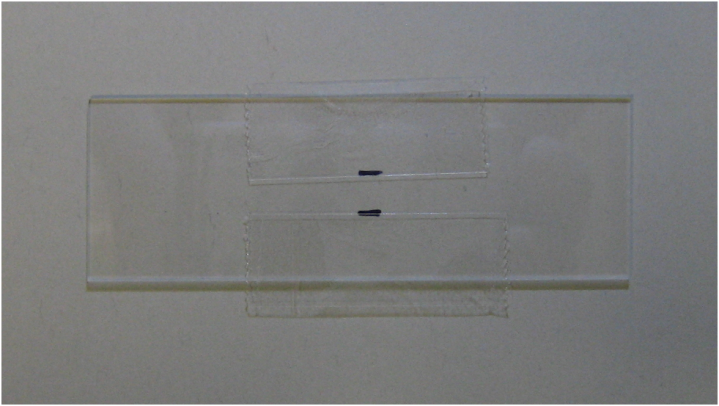


*Step 3:*

Excess tape is removed with the box cutters. The box cutters have spacers between them that are approximately the width of the cover slips. In order to prevent the tape from buckling while cutting off the excess, pressing the box cutter combo on the tape as shown in the image below helps. Be careful not to allow anything to touch the channel between the pieces of tape.


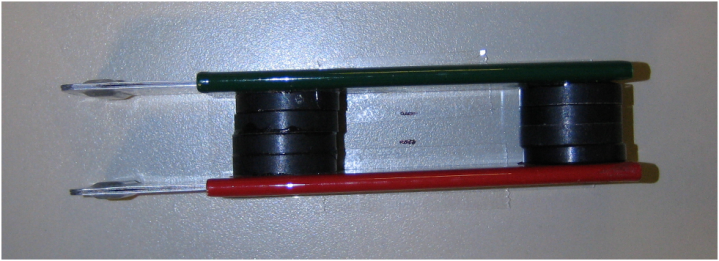


*Step 4:*

Once the excess tape is removed, a cover slip is placed over the tape channel. For proper adhesion to occur, the cover slip is pressed to the tape until the transparency of the glass+tape combo is uniform. This is easily done with a blunt object such as the end of one of the box cutters. Ensuring that the cover slip adheres to the flow cell properly will allow you to store flow cells over an extended period of time without worrying about their integrity.


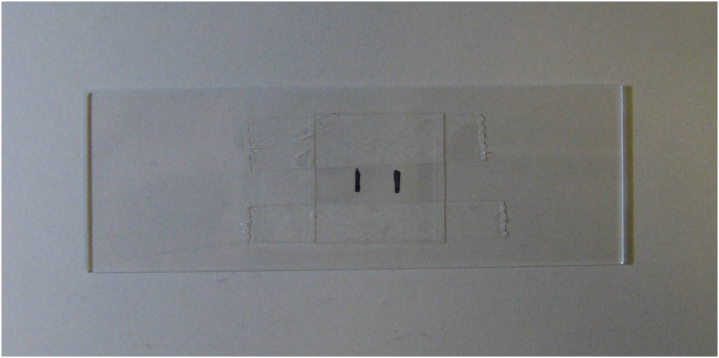


*Step 5:*

Exchanging the fluid in a flow cell is done by wicking away the fluid in the cell with a tissue.


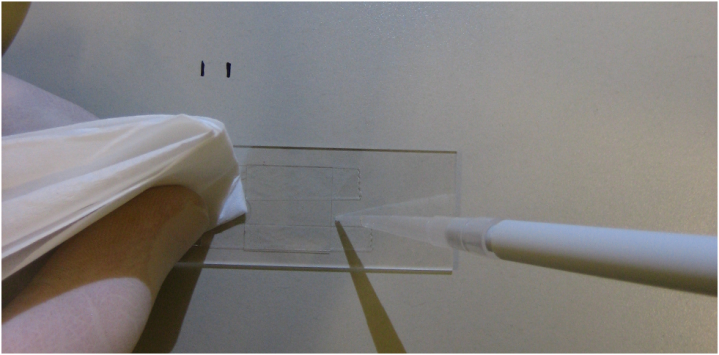


*Step 6:*

The final step before observation of the flow cell is to seal it with nail polish. We use nail polish made by NYC. We have not done experiments using other nail polish.


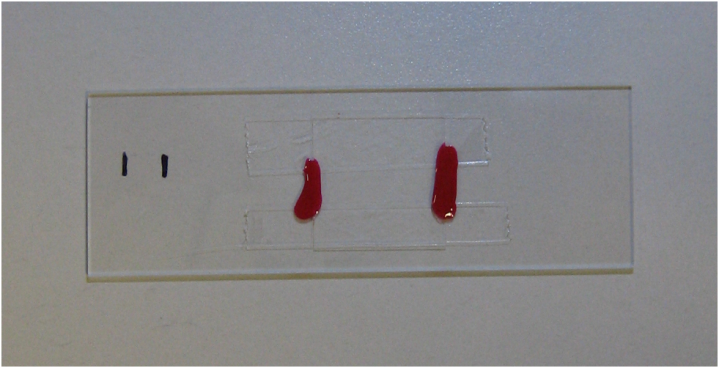

Supplement: Text S4 — Whole casein in PEM. Here we describe in detail how we dissolved whole casein in PEM. (DOC) [file pone.0019522.s004.doc]
